# Experiences of Transgender Women with Speech Feminization Training: A Qualitative Study

**DOI:** 10.3390/healthcare10112295

**Published:** 2022-11-16

**Authors:** Clara Leyns, Cassandra Alighieri, Jana De Wilde, Kristiane Van Lierde, Guy T’Sjoen, Evelien D’haeseleer

**Affiliations:** 1 Center for Speech and Language Sciences (CESLAS), Department of Rehabilitation Sciences, Ghent University, 9000 Ghent, Belgium; 2 Department of Speech-Language Pathology and Audiology, University of Pretoria, Pretoria 0028, South Africa; 3 Department of Endocrinology, Ghent University Hospital, 9000 Ghent, Belgium; 4 Center for Sexology and Gender, Ghent University Hospital, 9000 Ghent, Belgium; 5 Department of Otorhinolaryngology, Ghent University Hospital, 9000 Ghent, Belgium; 6 Musical Department, School of Arts, Royal Conservatory Brussels, 1000 Brussels, Belgium

**Keywords:** transgender voice, effectiveness, speech therapy, interviews, qualitative

## Abstract

This study investigated the experiences of transgender women after following sessions for speech feminization using semi-structured face-to-face interviews. Transgender women who completed a clinical trial were invited for an interview and 12 accepted the invitation. Interviews were conducted using an interview guide and were recorded and transcribed verbatim. NVivo 12 was used for qualitative data analysis, applying an inductive thematic approach. Four main themes were identified: communication, therapy experiences, impact on mental health, and external factors associated with the outcomes. For most participants, fear of speaking in public decreased after the training and all participants mentioned improved vocal characteristics. Though, reactions ranged from needing more speech therapy to being satisfied with the results. Coping strategies during misgendering occasions differed a lot between participants. More emotive counseling during speech feminization sessions is necessary to help clients in managing possible negative emotions.

## 1. Introduction

The terms trans, transgender, or transgender and gender diverse are umbrella terms that cover a wide spectrum of people who do not identify with their gender presumed at birth [1,2,3]. The development of an authentic, gender-affirming vocal presentation that matches their perceived gender is an essential aspect of the transitioning process for many transgender individuals [4,5]. The voice is an expression of self-identification and reflects the individual’s identity as well as gender perceived by others [6,7]. ‘Gender euphoria’, i.e., positive feelings about being affirmed as one’s true self and described as the opposite of gender dysphoria, can be experienced by transgender people who are able to express their gender through their personal presentation such as their voice and speech [8,9,10]. Transgender people might seek support from a speech-language pathologist (SLP) for voice modification [11]. During this speech training, transgender individuals are assisted in achieving their goal of voice-related gender expression [12].

Research on the quantitative effects of speech interventions in transgender women (presumed male at birth and a female gender identity) has been performed before and showed promising results [13]. Speech therapy could result in vocal changes, such as pitch elevation or a change in resonance, and gender perception [14,15,16,17,18,19,20,21,22,23,24,25,26,27]. The results of the included studies are sometimes difficult to interpret and compare due to methodological issues, such as small sample sizes and vaguely described therapy contents. Qualitative research may additionally reveal experiences unique to individuals within a training program [11]. One of the strengths of qualitative research is providing an understanding from participants’ perspectives about how they interpret or make sense of an event, situation, or action [28].

Stewart, Oates [28] explored transgender women’s experience and awareness of their vocal communication and voice use within sporting environments. They concluded through individual interviews that the voice is critical for transgender women when they wish to integrate and be accepted in different environments. Pasricha, Dacakis [29] conducted focus groups and aimed to gain an understanding of the way transgender women perceived their communication and their fulfillment in different aspects of their lives. They observed that transgender women find it difficult to maintain a feminine voice while they experience emotion or when talking to strangers. Moreover, the strongest amount of dissatisfaction with their voice was when talking on the telephone. Interviews and autoethnography were used to explore how the voices of transgender women were experienced in the study by Werier [30]. This was researched by paying attention to how we understand our own voices, as well as how and why we change our voices. The authors stated that voice matters most of all since it has a direct impact on the lives of many people, such as the difference between danger and safety, and recognition. The mixed-methodological study of Quinn, Oates [11] compared participant experiences in an intensive voice training program for gender diverse people aiming to develop a perceptually feminine-sounding voice. The experiences of the participants were compared using a satisfaction questionnaire as well as a thematic analysis of semi-structured interviews conducted with participants in the intensive voice training group. The results of this study suggested that the individuals in the intensive training program had both positive and negative experiences related to the intensive schedule, but that their preference was for the intensive training program. The semi-structured interviews conducted by Azul [31] focused on transgender men and subjective gender positionings concerning voice. Variable levels of satisfaction with vocal gender presentation and attribution were mentioned by the participants. In the qualitative study by Ahmed [32] where transgender individuals’ experiences on voice and technology were explored, participants expressed frustration on how rigid gender norms influence their experiences with voice, as well as their gender transitions more broadly. The participants explained during semi-structured interviews that their views on voice training were often complex and evolving. They stated that voice training can be both necessary and prescriptively limiting. An ideal design for a voice training application included an individualized and judgment-free goal setting, reflection, and actionable feedback.

Previous studies have shown that the collection and analysis of qualitative study designs are very meaningful and important to find perspectives from transgender clients that are unattainable through quantitative research. However, a prospective study investigating experiences of transgender women after a strict therapy protocol with an elaborate qualitative methodology following the Consolidated criteria for reporting qualitative research (COREQ) has not been executed so far. A randomized controlled trial (RCT) was organized with transgender women, consisting of 10 sessions of pitch elevation and articulation-resonance training. The participants of this RCT were invited for an individual semi-structured interview. By doing so, this study aimed to collect experiences of the voice feminization sessions and investigate their current communication and well-being.

## 2. Methods

This research project was completed according to the Declaration of Helsinki and approved by the Ethics Committee of the Ghent University Hospital with the following registration number: B670201941335. A written informed consent was signed by each participant.

### 2.1. Researcher Positions

Leyns C. (MS, SLP, PhD Candidate) is a cisgender woman (pronouns she/her) with 7 years of experience in voice and 4 years of experience in transgender voice. She has been researching the effectiveness of speech therapy for transgender women during her PhD. She is a member of a multidisciplinary gender team and the World and European Professional Association of Transgender Health since the start of her PhD in 2019. Her role on this project was conducting the RCT as the SLP who provided the therapy sessions, data analysis, investigator triangulation and manuscript writing.

Alighieri C. (MS, SLP, PhD) is a cisgender woman (pronouns she/her) with 7 years of experience in speech and 4 years of experience in qualitative research with multiple qualitative publications. She has 5 years of clinical experience in diagnosing and treating voice and speech disorders. During this project, she participated in the investigator triangulation (as researcher with a qualitative research background), manuscript writing and the interpretation of the data.

De Wilde J. (MS, SLP) is a transgender woman (pronouns she/her) with research experience in transgender voice during her master education. She is an elite vocal performer and she was part of the investigator triangulation (as SLP who can also advise from a transgender background).

Van Lierde K. (MS, SLP, PhD) is a cisgender woman (pronouns she/her) and a full professor in SLP. She has 30 years of experience in research and clinical voice work. She has been researching the effectiveness of voice therapy techniques. Her role on this project was defining the protocol of the qualitative research design, discussing the results and manuscript writing.

T’Sjoen G. (MD, PhD) is a cisgender man (pronouns he/him) with 22 years of experience in transgender health research. He has, as endocrinologist, acted as a principal investigator in the endocrine part of the ENIGI study (European Network for the Investigation of Gender Incongruence), he is a past president of EPATH (European Professional Association for Transgender Health) and is member of a multidisciplinary gender team. His role on this project was patient recruitment and manuscript writing.

D’haeseleer E. (MS, SLP, PhD) is a cisgender woman (pronouns she/her) and an associate professor in SLP. She is the promotor (senior investigator) of this research and has 16 years of clinical and research experience in voice and 6 years in transgender voice. She is a member of a multidisciplinary gender team and the World and European Professional Association of Transgender Health. She is responsible for the conceptualization and methodology of the research and was the interviewer of the transgender clients participating in this research. She was also part of the investigator triangulation, the interpretation of the data and manuscript writing.

### 2.2. Participants

Participants were recruited through the gender team of the Ghent University Hospital (Belgium) and participated in a speech feminization program [33]. Inclusion criteria for the RCT were an established diagnosis of gender dysphoria and female gender identity confirmed by the interdisciplinary gender team at the Ghent University Hospital (Belgium), age between 18 and 70 years, self-reported normal hearing, Dutch speaker, with gender affirming hormonal treatment (both estrogens and anti-androgens, or after orchidectomy estrogens alone), a female gender role and seeking voice feminization care. Exclusion criteria were: a history of neurological disorders, previous phonosurgery or voice and communication training to feminize the voice, organic pathology of the vocal folds, or smoking. 30 transgender women met these criteria and were involved in the RCT. For the current study, invitations were sent out for the interviews through email to the participants of the RCT and 12 individuals accepted the invitation. The age of the participants ranged from 22 to 58 years, with a mean of 33 years (SD: 10.9). 7/12 participants were in the group that started with articulation-resonance training (ART) and 5/12 started with pitch elevation training (PET).

#### 2.2.1. Speech Feminization Program of the RCT

##### Study Design

The program used a randomized controlled trial with cross-over design. Participants were randomly assigned to a group and received 14 weeks (15 h) of speech training. All participants started with 4 weeks of sham training after which they were randomly assigned to one of two groups: one group continued with 5 weeks of PET, followed by 5 weeks of ART and the second group received both trainings in the opposite order.

##### Speech Intervention

All participants received the speech training in a sound-treated room at Ghent University Hospital. The interventions were carried out by a certified and experienced SLP (CL). Sham training lasted for 4 weeks (5 h), 1 session of 75 min per week, and included discussing vocal hygiene, anatomy, voice characteristics, non-verbal communication, relaxation, and breathing exercises. Both the PET and ART lasted for 5 weeks (5 h), 1 session of 60 min per week. A detailed description of the sessions of PET and ART can be found in Appendix A. Participants received a homework chart (Appendix B, Table A1) where they could indicate whether they practiced or not. They were encouraged to exercise twice a day, 10 min each.

### 2.3. Data Collection of the Interviews

Semi-structured interviews were conducted in Dutch in a face-to-face setting. As the first author was the therapist during the speech feminization training, the authors decided that another SLP with experience in transgender voice should conduct the interviews to avoid bias. The interviewer (ED) was a qualified SLP and researcher who was experienced in the field of voice for more than 16 years and 6 years in transgender voice. All interviews took place in the speech lab of the Ghent University Hospital and were recorded with a LifeGoods digital voice recorder and lasted 42 min on average (SD: 14.2, range: 25–76). Data saturation was reached after 10 interviews and thus 2 extra interviews were performed to validate the saturation. These interviews did not produce any new codes, which validated the data saturation.

An open question was used to start the interview: “How did you experience the speech therapy sessions for speech feminization?”. Then, the interviewer continued with different topics that were introduced by the participant. An interview guide was designed by the authors (Appendix C, Table A2) which included topics such as general communication, voice before/during/after the speech therapy sessions, effects and expectations of speech training, communicating with others, misgendering and mental impact. This guide was used to bring up new topics when the interview stagnated or when additional information was needed. Follow-up questions were used to explore themes that were mentioned by the transgender women.

### 2.4. Data Analysis

The data analysis was performed with the qualitative analysis software program NVivo 12 [34,35,36]. After recording the interview, verbatim transcriptions were made by CL. All the identifiers of the participants were deleted to ensure anonymity during the coding process. The collected data were analyzed to discover common and recurring themes [37] through labeling quotes in the interviews, i.e., a thematic analysis. Thematic analysis is a method for identifying, analyzing, and reporting patterns within data, i.e., themes [38]. It minimally organizes and describes your data set in detail. However, the analysis interprets various aspects of the research topic [39]. Thematic analysis is compatible with both essentialist and constructionist paradigms. Through its theoretical freedom, thematic analysis provides a flexible and useful research tool, which can potentially provide a rich and detailed, yet complex account of data [38]. An inductive or ‘bottom-up’ approach [40], rather than a theoretical or ‘top-down’ way [39,41], was used in this study. An inductive approach means the themes identified are strongly linked to the data themselves [42]. Therefore, the themes would also not be driven by the investigator’s theoretical interest in the research topic. Inductive thematic analysis is a process of coding the data without trying to fit it into a pre-existing coding frame. Consequently, this form of thematic analysis is data-driven [38]. The 6 phases of Braun and Clarke [38] were used in the current study. Phase one is ‘familiarizing yourself with the data’. The author read and re-read the data, transcribed the data, and noted down initial codes. Phase two is ‘generating initial codes’. Interesting features of the data were coded in a systematic manner across the dataset, organizing data relevant to each code. The third phase is ‘searching for themes’. Codes were assembled into potential themes and all the data relevant to each potential theme were gathered. Phase four is ‘reviewing potential themes’. In this phase, the authors checked whether the themes worked in relation to the coded extracts and the entire dataset. A thematic ‘map’ was generated. Phase five is ‘defining and naming themes’. Specifics of each theme were refined, and a clear name was generated. The sixth phase is ‘producing the report’. A report was produced, and exemplar quotes were provided in the results section to support the different themes [43,44]. The Dutch quotes were translated into English based on a consensus between all the authors. The Consolidated criteria for reporting qualitative research (COREQ) were used to report the study [45].

### 2.5. Trustworthiness

Methods to ensure the trustworthiness of the data were implemented in this study, such as the inclusion of investigator triangulation. Doubts around certain themes or codes found in the transcription of the interviews and the actual meaning of the data were discussed. By doing so, the credibility and confirmability of the findings were safeguarded [46]. Four individuals were included in the investigator triangulation: CL, the therapist during the trial with clinical and research experience in the field of transgender voice, ED, the interviewer with experience in the field of transgender voice, JDW, an SLP with a transgender background, and CA, a researcher with experience in qualitative studies. The first author and coder (CL) developed a personal reference framework which was given to the other researchers before discussing the triangulation. A member check was performed during the interviews to increase the trustworthiness of the data [46]. The interviewer restated and summarized the information of the participants to determine the accuracy of their responses. An audit trail, providing a thorough and transparent description of the research process, the data collection, analysis, and the rationale of meaning assigned to the data, increased the confirmability and dependability of the results [46,47].

## 3. Results

Four main themes have been identified: therapy experiences, communication, impact on mental health and external factors associated with the outcome. Several subthemes have been identified as well and are more thoroughly described below. A table with themes, subthemes and selected quotes has been added in Appendix D (Table A3).

### 3.1. Therapy Experiences

People described the therapy sessions as being interesting and fun, clearly explained, very positive, really good, comfortable, effective, educational, surprising at times, and structured. They felt that they were working on themselves. Participant 8 brought up that the training was a mixed process for her. As following speech therapy sessions was something she had not done before, she sometimes felt uncomfortable and out of her comfort zone making strange sounds in front of somebody. Concerning the duration of the training, nobody said that they found it too long. Some said that they received all the tools that they need to continue on their own, others said that they needed more time and more sessions. Especially the articulation-resonance training was considered to be intensive:

“*Especially the part with the clear speech exercises, it was always 1 topic per session and then actually that last session was everything together, and that was all quite short together. A bit too difficult, I had also practiced the other (techniques) a bit, but only practicing one item at a time and then having to apply it again after 5 weeks and add the last one, was quite fast. Way too many (techniques). I thought the pitch part was a good tempo, I thought that was better*.”

*(Participant 12)*

“*I sometimes had the feeling that it was going a bit fast at times. But it was good that most themes got their own session at the very least. Uh yes, it’s also the fact of, I have no experience with music or with voice in any other way, uhm, so for me that was all new information, uhm and because of that I sometimes had the feeling, yes, that it sometimes went quite fast or I found it difficult to hear correctly, how a certain technique affected the sound of my voice*.”

*(Participant 3)*

Another person found the intensive training good for her as she felt that the connection with the therapist was better compared to having long breaks in between sessions and that the exercises were more natural for her because of that. The weekly appointments were perceived as appropriate, as they did not want too much time in between the sessions. One person mentioned that the distance for commuting to the clinic was too big. Four subthemes have been identified in the therapy experiences theme: voice changes, difficulties in the training, relationship with the therapist, and home exercises.

#### 3.1.1. Voice Changes

Higher pitch was the most described voice change. More rising intonation patterns, clear articulation and resonance, i.e., using head and forward resonance, lip spreading, were changed as well. Being more in control of their voice and knowing how to have good vocal hygiene was also noted by some. Though, being more self-conscious about their voice was also an aspect that was mentioned.

Concerning the expectations of the therapy sessions, most people did not expect anything at the start of the training. Participant 6 did not have any expectations as she thought that she would not be able to succeed.

“*Pretty low, because I know my voice is pretty low. Eh and I’ve always had problems with that so I kind of had the idea of speech therapy, I wanted to try it, but personally I had little expectations. That had nothing to do with whether it was speech therapy or not, rather because I know I have a low voice*.”

*(Participant 11)*

Six persons looked up some information on how to change their voice, but were afraid to strain their voice and wanted to have an expert opinion. Another person said that she knew that after 14 sessions (of which 10 were speech feminization) she would not have the most perfect voice, but that it would be a first step and that this was enough for her. She said:

“*I also don’t need to have the highest, not the most ‘fem’ voice that… Uh if I really wanted to I probably could, I do have the voice range for it, but it’s not really necessary for me. I already have something like, I’m where I want to be, and that was actually what I wanted, actually more than I wanted from the study*.”

*(Participant 12)*

Reactions on whether their expectations were fulfilled after the training varied between being happy with the training and exercises that they received and were supported by an expert, to being dissatisfied with the results but blaming themselves, i.e., being too stressed, caused by underlying ADHD, ASS, or by not practicing enough.

“*So at the end of the sessions I was really impressed by the big difference between before and after. I actually came in with pretty low expectations and I came out with very satisfied results*.”

*(Participant 5)*

“*Ehm, yes, since I haven’t practiced much on myself yet, that hasn’t come up much yet, but that’s up to me. Not on the study, that’s just myself, who was under too much stress*.”

*(Participant 1)*

Some did not reach their ultimate goal of achieving a voice which is in line with their gender identity. For participant 10, her voice sounded unnatural to her. She still wanted to work on her pitch as she was able to achieve good results with her resonance. Reaching a consistent pitch in daily life communication was a hurdle experienced by seven participants. Some experience fatigue when trying to keep a higher pitch. Another person wants to change her intonation patterns. When asking whether they needed more speech therapy sessions, reactions were mixed as well. Some thought they needed more sessions, some said that they would benefit from more sessions but that it was not a priority right now, and others thought they had the right tools to practice more on their own if they thought that was necessary. Participant 1 considered doing phonosurgery but she was afraid of the complication of hoarseness. For another person, surgery was one of the next steps, as she thought that it would reduce her speaking anxiety, and that it would help her feel ‘fully feminine’.

#### 3.1.2. Difficulties in the Training

When the different exercises were discussed, five persons found lip spreading exercises the easiest to use in their speech, four people said that they found the forward tongue position an easy exercise. Two persons mentioned they had no trouble at all during pitch elevation. Only one person mentioned ‘everything’, ‘clear speech’, ‘spontaneous speech’, ‘twang’ and ‘intonation’ as an easy exercise. Difficult exercises were pitch elevation (n = 4), twang (n = 4), forward resonance (n = 3), water resistance therapy during pitch elevation, clear speech, intonation, forward tongue position, or nothing (n = 2).

A lot of participants (n = 7) acknowledged that the generalization of the techniques was an additional difficulty. When they were not focusing on the technique, their voice changed again after a few sentences. They addressed the fact that it was tiring to constantly think about it. Participant 7 mentioned that she could easily put that into perspective.

“*And if I notice that I forget to apply a technique in a conversation, I can also think about it for a while that I forgot to apply it. But then I just think, next time we’ll do it better*.”

*(Participant 7)*

Participant 6 said that shouting was quite impossible to do with a higher pitched voice, and participant 11 experienced her voice could be quite hoarse when speaking. She mentioned that she exploited that hoarse voice as some kind of mask, to cover up the fact that she thought she spoke with a low pitched voice.

“*Uhm, well, to be honest I try to use it as a kind of mask for my low voice, so if I’m not hoarse then I actually have a very low voice, but if my voice is as hoarse as it is now then I use that in actually to cover up my low (voice), so yes, so for example now with a relative, in-laws, and I say there’s something wrong with my voice, and then she says yes that’s normal, but that’s actually to cover up that I have a low voice*.”

(Participant 11)

#### 3.1.3. Relationship with the Therapist

Six out of the twelve participants mentioned having a good relationship with the therapist were very beneficial for their progress. They liked having one-on-one sessions. Receiving feedback from that person, being motivated, and being guided patiently, step by step, was very important for them. Participant 5 felt that she was not only supported on speech level, but appreciated the times when counseling was at hand, and the therapist was available on an emotional level.

“*That’s something where the therapist really helps, that you stay less focused on that bigger (goal), because that, that’s a bit like you want to run a marathon when you’re at a point where you can’t even run 5 km. There are still many intermediate steps to take and if you can’t see them it feels very intimidating and very unreachable*.”

*(Participant 3)*

#### 3.1.4. Home Exercises

Not everybody had good experiences with practicing at home. Participants 2 and 3 said that they enjoyed the first home exercises but found that after a few weeks, they lost motivation and started to practice less consistently. Others had very busy personal/work and speech therapy was not something to easily fit into their schedule. Some mentioned that they were very stressed, or forgot to practice (n = 3).

“*Uh, yeah, I just forget to do them that’s my problem. It’s just, I’ve already got so many other things to do that I’m just not going to get around to it I guess.*”

*(Participant 1)*

Participant 2 did not like the repetitive element in the lip spreading and forward tongue position exercises.

“*In the beginning, because, yeah talking with lip spreading, yeah and with that forward tongue, that, I don’t know, that was exactly the same all the time in that bundle (of exercises), then I didn’t really think that…, that was so very repetitive and always the same and I wanted to bring a little more variation in that so I tried to practice that more by simply applying it more in daily life, for example by going for a walk with my brother and then trying to pay attention when I was talking*.”

*(Participant 2)*

Participants 3 and 5 were really motivated to practice a lot at home, confronting their dysphoria and feeling that the sessions gave them some kind of direction in ‘going forward’. Some refused to do the exercises at home, but tried to implement them directly in their daily speech.

### 3.2. Communication

General aspects of communication were mentioned in this theme. Fear of speaking in public was something that was mentioned by ten participants. Some would avoid talking to strangers, as they were afraid to be misgendered or because they were ashamed of their voice. For some people this had changed after the training, as they felt less inhibited to communicate with people. Participant 7 did not telephone people and communicated everything through email as she was scared to be misgendered. However, after training she did start calling people again. Both participant 3 and 10 mentioned that they were more sociable after therapy, and that they dared to talk more to strangers and communicate their feelings. Participant 2 and 10 on the other hand found it still difficult to talk to people. Participant 1 said that written communication was easier than verbal communication, but that she will say more when it was face-to-face. Participant 11 pointed out that she still avoided talking in public, she would rather communicate non-verbally and use hand gestures instead of using her voice. Two subthemes emerged from the communication theme: priority of the voice and use of the new voice after therapy.

“*Yes, I may feel happier because I don’t have to hold back. For example those phone calls, instead of an email that I can now really call directly and that I don’t feel bad about it. That is a bit of happiness* <<laughs>>. *So actually before the therapy I didn’t really have a fear of speaking in daily life, I wasn’t really afraid of it, but they had to see me. And now I dare to have more and more conversations without them seeing me. Like in the past when a friend who called me or something, I declined it and sent a message instead, that was so bad. And now I’m calling right away. That’s a difference.*”

*(Participant 7)*

#### 3.2.1. Priority of the Voice

On the one hand, participants felt that their voice was very important in their transition and their life in general. Their voice could be a main obstacle in their transition, even when they were fully satisfied with the rest of their physical, social, and psychological transition. On the other hand, their voice could also feel as an ‘extra’ thing, but not the most important part of feeling content. Two individuals mentioned that going to other healthcare providers such as endocrinologists, surgeons, psychologists, beauticians, etc., was already a lot to plan in their daily life and that their voice, and following speech therapy, was not a priority in that sense.

“*It is certainly not a frustration of mine, in the end I feel good, I have lived in hell and now I enjoy life. The voice is then, I’ll just say ‘surplus’. But it is certainly not a must*.”

*(Participant 9)*

#### 3.2.2. Use of the New Voice after Therapy

Some participants noticed that at the time of the interview, it was difficult to use their ‘old’ voice because they were accustomed to their ‘new’ voice. For them, the changes went quite smoothly. Participant 7 motivated herself that she would be able to do ‘it’ next time, after she noticed in a conversation that she was not using some of the techniques she learned. On the other hand, some participants experienced that continuously using their new voice was challenging, especially in a conversation after a few sentences. Participant 5 did not dare to use her new voice in her environment because she wanted to practice more on her own before applying it in a real-life situation with family or friends.

“*And now just the other day somebody said, “Say something in your old voice again, because I can’t remember.” And that didn’t work, I couldn’t sound the way I used to. It has really become such a habit, even to the kids and everyone who knows me, I just keep using a higher pitch*.”

*(Participant 6)*

Most participants changed their voice depending on their communication partners. When they were talking to family or close friends, they feel more relaxed and subconsciously use their old voice again. During short conversations with strangers, they generally find it easier to use their preferred voice. When the topic arose whether their new use fits their expression and identity, participant 10 wanted to highlight that the idea of being passable was never the ultimate goal, but feeling happy about herself was, which was also acknowledged by participant 9. She now achieved this goal because of the sessions. However, two other participants said that they were not satisfied with their voices.

### 3.3. Impact on Mental Health

Most participants found it hard to determine to what extent the sessions had an impact on their mental health. Two participants mentioned that they were going through a lot of other difficulties that were not related to the voice.

“*I don’t actually know. I do not know. Because there are ten, twenty other things that also play a role that I really just don’t know anymore. My voice probably helped a lot with that, but (..) yes, I think it’s difficult to estimate how big the share was*.”

*(Participant 1)*

Participant 12 described it as two-sided.

“*Um, it has two parts. One, feeling good in what I am and I’ve always had that. I think my voice is more beautiful now than before, but I also thought my voice was beautiful then, it just didn’t quite match what I was and how I felt. Because I was different then than I am now. But on the other hand, voice is also something that is judged a lot, one of the first things people see/hear about you and one of the first things people give a first impression, and if you can make that first impression positive that is a boost for yourself as well. And do you feel better about that, especially if it also has a gender-affirming effect.*”

*(Participant 12)*

Two other participants acknowledged that when they were in a bad mood, their voice would have a bigger impact on their mental health, than when they were in a good mood. Participant 2 was sometimes jealous when she heard other voices in public. She experienced depressive thoughts when she started to focus on her voice.

“*Uh, I don’t know, sometimes that gives me a hopeless feeling that I have the impression that I am still very far away, if I can compare it with an analogy it is exactly that as a child you are playing with blocks and then that after a lot of training you can place three blocks on top of each other, but when you see that other people can immediately place ten blocks on each other, then you are not happy at all with the progress you have made of putting those three blocks together. Then you can only focus on ‘I’m really not quite sure what I would like to have’. And yes, that’s how it feels.*”

*(Participant 2)*

When talking about the impact on their mental health, participants mentioned the impact on their self-confidence as well. Everybody said that they had improved self-confidence. One person on the other hand commented that it was two-sided.

“*On the one hand I may have the impression that I have become a bit more insecure because I don’t know what I sound like at all, but I think that’s because I used to know I sound like a man and then I don’t care anymore and I was also less involved with it so, because I’m more involved with it now, I’m going to worry about it a little more often. Then on the other hand, yes then I also feel better that people think they think I sound more feminine, or more in between, and um, yes I don’t know, in that respect I think I should actually feel more confident, but I don’t always feel that way.*”

*(Participant 2)*

A subtheme around misgendering also emerged from the data.

#### Misgendering

Some participants were still being misgendered in public, especially on the telephone. Participant 11 said that the only way that people still misgender her, was because of her voice. Eight persons noticed that misgendering occasions significantly decreased since the start of their sessions. Very negative feelings were brought up by participants 3 and 4, who felt frustrated, sad and had feelings of shaming and blaming themselves when misgendering occurred.

“*Uh (..) yes, so (...) it’s a bit of frustration, a bit of sadness, so what the feeling of, yes, falling short, yes, partly with that I’ve been working on it for a while, is it so a bit like, will I never get there, yes. Yeah, kind of like that kind of feeling*.”

*(Participant 3)*

“*Bad feelings, I feel really really bad about that. Yes, it is, yes, how would I say, a knife you get in your own back, that hurts a lot. That hurts a lot.*”

*(Participant 4)*

The coping strategy can differ per person, and usually depends on who their communication partners are.

Not everybody reacts when somebody misgendered them. Some said that it depended on how they were feeling at that moment whether they would correct the other person or not. Participant 12 thought that society had become more tolerant in gender expression:

“*I think that’s also because people are also a bit more free in their gender expression these days, euh, which sometimes makes people think ah that’s just someone who is a very feminine man or something. And uh, which is also positive on one side. So I’m like, as long as it’s said kindly, and like, ‘oh sorry’, I don’t have a problem with that. It’s not something I think about afterwards or anything, it’s not something that invalidates me*.”

*(Participant 12)*

Participant 6 said that she just let it pass whenever misgendering happened. She addressed the fact that also cisgender women get misgendered sometimes because of their lower voice.

### 3.4. External Factors Associated with the Outcome

For nine participants, their voice changed depending on who their communication partner is. When they were really comfortable with them, they mostly had a lower pitched voice compared to talking with strangers. Participant 12 said that she used a more feminine voice when talking to strangers, as she believed that for people who knew her better, the voice was just a part of her as a person. Participant 9 reported that she blamed herself when she did not use her higher pitched voice when she was talking to strangers, even though she was actually content with the voice she used in daily life.

Reactions from friends and family on their voice also seemed to be important. For people who received positive feedback from others, they felt supported. Participant 1 mentioned that she got the impression that people did not really pay attention to these changes, and maybe they did not notice it because it went slowly. Participant 4 on the other hand, noted that her partner sometimes laughed at her, and that this made her insecure about doing the exercises. Other participants did not get feedback from people around them.

Concerning work, participant 3 noticed that she was more open now to job opportunities that need some kind of client interaction, which was something that she never thought to be possible. Working in a loud environment can hamper the possibility to practice during work.

## 4. Discussion

This study invited transgender women, who were enrolled in an RCT including 10 speech feminization sessions, for an individual semi-structured interview. The goal was to investigate the experiences of these transgender women about the sessions, their communication and their well-being. Four main themes emerged from the semi-structured face-to-face interviews: communication, therapy experiences, impact on mental health, and external factors associated with the outcome. From these themes, several important aspects were discussed below.

### 4.1. Satisfaction after Speech Training

Everybody improved in their vocal aspects. Some mentioned that their pitch was easier to change, others preferred resonance characteristics. Everybody found the training an enjoyable way to change their vocal characteristics. The degree to which they were satisfied varied and sometimes depended on personal or external factors. These results were in line with the qualitative study by Azul [31], where variable levels of satisfaction with vocal gender presentation were described by the participants. Another qualitative study found through semi-structured interviews that the participants’ views on voice training were complex [32]. In general, those who are not misgendered anymore in the current study were most satisfied after the training, and vice versa. Furthermore, the well-being of the majority of participants seemed to depend mainly on the way in which they dealt with the misgendering. Concerning the duration of the protocol, nobody mentioned that the program was too long, on the contrary, some individuals said that it should be longer, as the program was quite intensive, learning a different technique every week. Quinn, Oates [11] also found that experiences in intensive training programs may be highly variable and mediated by factors such as clients’ individual personalities and preferences. Additionally, some people who thought they learned about all the necessary aspects to feminize their voice and had the tools to further improve their voice if they want to.

### 4.2. Need for Generalization

Most participants in this study agreed on the fact that the generalization of speech feminization techniques was still difficult. Using their feminized voices in longer conversations, but also in different speaking situations is crucial. The communication partner was also important, as they found that using the techniques with persons that they are generally comfortable with, such as close family and friends, is harder than using their feminized voice with strangers. These results are in line with the acoustic outcomes of this trial, as the lower limit of speaking fundamental frequency during spontaneous speech, which is important for gender perception [48], did not increase [33]. Ten hours of speech feminization might thus be too short for a complete generalization of the used techniques and should be further investigated in future research.

### 4.3. Emotive Counselling

Different coping strategies were identified from the interviews. Some people who let negative interactions, such as an occasion of misgendering, pass by. They relativize the event by recognizing that not only transgender people can be misgendered. Cisgender women with lower pitched voices can be attributed as a man as well. However, for some individuals, the impact of misgendering is very high. It can impact their mental health for a long time. Even strong feelings of self-blame and shame emerged from the interviews. People mentioned that it was their own fault and that they did not practice enough. Additionally, some participants changed their voices in order to avoid negative reactions from the outside world, even though they were happy with their current voice. Although the fear of speaking in public decreased for most participants, speech therapy increased this fear for one participant. She mentioned that before therapy, she was certain that people would gender her as a man, and after therapy, the uncertainty of that gave her more fear of speaking in public. It appeared that strangers’ views and opinions are very important for them, either in the gender attribution or the view of society on the meaning of gender expression. These aspects raised the need for more support of negative feelings during and after speech therapy. This could be explained by the specific therapy content of this trials, which was focused on pitch and resonance. In the study by Azul and Hancock [49], reconceptualization of clinicians’, speakers’, and listeners’ agency as bioculturally mediated capacity to act implied a lack of control over the production of voice and speaker socio-cultural positionings in vocal encounters. Researching a client’s perspective on how they perceive their voice and how listeners perceive their voice, might help the SLP to better counsel about the unpredictability of voice production and speakers’ socio-cultural positioning in daily life conversations [49]. Additionally, the SLP can help with developing confident responses to misattributions from conversation partners, as their psychosocial well-being is affected by these types of social and emotional stressors [49]. The findings in the current study confirmed that merely focusing on voice-technical aspects such as pitch and resonance is never enough and that the SLP should spend enough time on counseling. Having a good relationship with the SLP, which was also stressed by a lot of participants, can benefit these strategies as well.

### 4.4. Limitations

One of the limitations of this study was a possible participant selection bias. People willing to participate could be the participants who were all satisfied or the opposite, that people who are not satisfied can also feel the need to talk about their discontentment. Reasons for refusal of the interview were not collected either. The participant selection bias also made it hard to generalize these results. A clinical setting is different from an experimental trial, i.e., in an RCT, the therapist is bound to the content of the therapy sessions protocol and is not as flexible as during a very individualized clinical setting. Thus, generalizing the results is difficult once again. Additionally, the persons in this trial did not have any expectations about speech therapy. These individuals could have different expectations and motivations than people who search help from a private practice SLP. The low expectations can also be a protection mechanism, caused by previous disappointments in their preceding transition process, or just the fact that they had never done any speech training before the start of the program.

### 4.5. Innovativeness

Qualitative research may reveal experiences unique to individuals within a training program [11]. It can provide different understandings from participants’ perspectives about how they interpret or make sense of a situation [28] and it can therefore help a healthcare provider or multidisciplinary team to re-evaluate care pathways. Per the authors’ best knowledge this is the first study to include a strict qualitative methodology design, following the COREQ guidelines [45] and observing both therapy experiences, general communication aspects, mental health and external factors.

## 5. Conclusions

Overall, positive experiences regarding the training were observed during the interviews. Regarding the content of the voice therapy, not every voice technique worked for everybody and treatment goals should be individualized. A period of 10 sessions is too short to generalize speech feminization techniques in daily life. Future research should include more emotive counselling during the sessions to handle negative emotions or external factors such as misgendering occasions, as well as strengthening the transgender person in their self-confidence of using the voice that fits their gender identity and expression.

## Appendix A

**Therapy program**

1. **Pitch elevation training**
  a. Session 1
    i. Auditory descrimination with a piano
    ii. Glissando patterns (using biofeedback real-time pitch of Computerized Speech Lab (Kay Elemetrics)
      1. From habitual ‘old’ pitch to the ‘new’, higher pitch in isolated nasal consonants (approximately till 160 Hz); from habitual old pitch to highest pitch, etc.
    iii. Adding consonant-vowel-consonant combinations
    iv. Explanation of biofeedback tool to use at home: smartphone app Voice Pitch Analyzer
  b. Session 2
    i. Repetition of glissando patterns
    ii. Automatic sequences (counting from 1 till 10, days of the week, months of the year, etc.) starting on habitual old pitch and then on the new pitch
    iii. Automatic sequences with gliding from old to new pitch within the word
  c. Session 3
    i. Short warm-up with glissando patterns
    ii. Speaking with the new pitch, making sure there are a lot of upward intonation patterns
      1. Short expressions (e.g., ‘Go away’, ‘Mum and dad’, ‘up and down’, ‘be careful’, etc.)
      2. Building up sentences
        Can you
        Can you put
        Can you put those files
        Can you put those files in the storage room later on?
      3. Short sentences
      4. Poems
      5. Texts
  d. Session 4
    i. Introduction of water resistance therapy, using a resonance tube (2 cm under water)
      1. Bubbeling without phonation
      2. Phonation with old pitch in the tube
      3. Phonation with new pitch in the tube
      4. Glissando patterns in the tube
      5. Short sentences with new pitch in the tube, then without tube
      6. Poems with new pitch in the tube, then without tube
      7. Texts with new pitch in the tube, then without tube
      8. Spontaneous speech, answering in the tube, then without tube
        a. Short answers (1 sentence)
        b. Longer answers (2–3 sentences)
        c. Conversation
  e. Session 5
    i. Repetition of water resistance therapy
    ii. Straw phonation
      1. Blowing without phonation
      2. Phonation on new pitch
      3. Glissando patterns
      4. Spontaneous speech, answering in the straw, then without straw
2. **Articulation-resonance training**
  a. Session 1
    i. Lip spreading
      1. Alternating with making an /u/ and /i/ movement of the lips (= discrimination with lip protrusion and lip spreading). Using a mirror to look at the lip movements.
      2. Alternating with making an /u/ and /i/ sound.
      3. Alternating with making an /e/ and /y/ sound.
      4. Consonant + /i/ combinations, consonant + /e/ combinations, feeling the easy lip spreading
      5. Trying to reach lip spreading when doing consonant + /u/ and /y/ combinations
      6. Monosyllable words with /i/, /e/, /u/ and /y/
      7. Multisyllable words with /i/, /e/, /u/ and /y/
      8. Sentences with /i/
      9. Sentences with /e/
      10. Sentences with all combinations
      11. Text
      12. Spontaneous speech
b. Session 2
  i. Repetition of lip spreading
  ii. Forward tongue position
    1. Awareness of the tongue muscle: non-speech oral motor exercises. Using a mirror to look at the tongue movements
    2. Moving the tongue from front to back when producing vowels
    3. Pronouncing /i/ (high vowel) and feeling the forward tongue position with a high back of the tongue
    4. Starting from /i/ sound and gliding to other vowels, trying to reach forward tongue and high back of the tongue
    5. Words with /i/ (high vowel)
    6. Words with /y/ (high vowel)
    7. Words with /a/ (low vowel)
    8. Sentences with /a/
    9. Words with /o/ (low vowel)
    10. Sentences with /o/
    11. Texts
    12. Spontaneous speech
c. Session 3
  i. Repetition of forward tongue position
  ii. Larynx elevation through twang
    1. Awareness exercise: yawning (downward movement of the larynx) and swallowing (upward movement of the larynx)
    2. Listening to twang sound such as crying baby, goat sounds, etc.
    3. Adding twang to vowel /a/
    4. Decreasing twang to vowel /a/
    5. Consonant + /a/ + consonant + /a/ + consonant + /a/ with twang
    6. Words with /a/
    7. Sentences with /a/
    8. Texts
    9. Spontaneous speech
d. Session 4
  i. Repetition of larynx elevation
  ii. Forward resonance
    1. Discrimination between chest resonance and head resonance, saying /o/ vowel
    2. Putting a finger on left and right nostril and saying ‘hmmm’, feeling forward airflow
    3. Nasal consonant /m/ + vowel
    4. Words with initial /m/
    5. Extra exercise to feel forward resonance
      a. Stand in front of a wall about 50 cm away.
      b. Place your head against the wall, comfortably.
      c. Your arms hang loose by your side.
      d. Place your tongue on your hard palate and start with a ‘nnnn’ sound. Make a few glissandos to high and low frequencies.
      e. Repeat the previous step but now place the back part of your tongue on your soft palate. Make a ‘ng’ sound and a few glissandos. By placing the head against the wall you feel the resonances better in your head.
  iii. Clear speech
    1. Combinations of consonants and vowels, pronouncing slow and then very fast, trying to pronounce clearly and precisely
      a. Tippetiptiptip tappetaptaptap toppetoptoptop
      b. Tanatanta tenetente tinitinti tonotonto
      c. Prieke prokke prakke pro prieke prokke prakke pro
      d. ...
    2. Word combinations, 3x slow and 3x fast
    3. Cork exercise: using a cork with a diameter of 23 mm and length of 45 mm.
      a. Placing the upper front of the cork (approximately 2–3 mm) between their front teeth and reading words out loud with large and precise articulation movements. After a block of long nouns (6–9 syllables), they removed the cork and used the same large articulation movements to pronounce the same block of words.
      b. Tongue twisters with and without cork
      c. Text: reading sentences with and without cork
    4. Spontaneous speech
  e. Session 5
    - Repetition of all articulation-resonance techniques, spending most time on forward resonance and clear speech
    - Generalization of all articulation-resonance techniques
      1. Texts
      2. Spontaneous speech

## Appendix B

**Table A1:** Homework Chart.

	Oefenschema (Homework Chart) Teken Een Smiley Bij De Dagen Wanneer Je Geoefend Hebt. *(Draw a Smiley/Circle/Cross on the Days that You Practiced) Ideaal: 2x Per Dag Telkens 10 min. (Ideal: 2x a Day for 10 min Each)*						
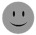
	Maandag *(Monday)*	Dinsdag *(Tuesday)*	Woensdag *(Wednesday)*	Donderdag *(Thursday)*	Vrijdag *(Friday)*	Zaterdag *(Saturday)*	Zondag *(Sunday)*
**Week 5**							
**Week 6**							
**Week 7**							
**Week 8**							
**Week 9**							
	Maandag *(Monday)*	Dinsdag *(Tuesday)*	Woensdag *(Wednesday)*	Donderdag *(Thursday)*	Vrijdag *(Friday)*	Zaterdag *(Saturday)*	Zondag *(Sunday)*
**Week 10**							
**Week 11**							
**Week 12**							
**Week 13**							
**Week 14**							

## Appendix C

**Table A2:** Interview Guide (English Version).

Topic	Questions
**Introduction**	**Introduce yourself** **Practical matters related to the interview:** - Thank you for participating in an interview. - Explanation on topic: “This part of the study will be about the experiences of transgender women about speech therapy. For this research, several interviews will be conducted with different people.” - In this interview we especially want to listen to your experiences. You cannot give wrong answers. - This interview is completely anonymous, we will not use or mention your name and personal details when processing the data. Everything you say will only be used for this research. - The interview will last approximately 60 min. You may indicate at any time during the interview if you do not wish to proceed. At that point we end the interview. - The interview will also be recorded later, this way we can fully focus on the conversation.
**Start interview**	I will start the interview with a very broad open question: *“How did you experience the speech therapy sessions that you followed for speech feminization?”.* This is a broad question, please take your time to think about it.
**Additional questions:** **Participant says X…** - **Can you tell us more about that?** - **Can you give an example of X?** - **You just said X, what do you mean by that?** - **You say you feel X, what do you mean by that?** - **Can you explain that a bit further?** - **How do you deal with that?** - **Did I understand correctly that…?** - **What did this mean to you?** **Remark:** **During the interview, non-verbally stimulate, paraphrase, summarize and ask for clarification.**	
**New themes are introduced depending on the participant’s answer.**	
**General communication**	**What does your voice mean to you?** Secondary question: How important is the way your voice sounds to you? **What does an adjustment to your voice mean to you?** Secondary question: How important was an adjustment of your voice to you?
**Voice before/after/during speech therapy**	**How has your voice changed since speech therapy?** **What impact did the sessions have on your voice control?** **What impact did the sessions have on your voice awareness?** Additional questions: - You mentioned that your voice changed, what changes did you notice? Which changes are important/less important to you? In what situations do you notice these changes? - You mentioned that you had certain voice complaints, what were these? - You mentioned that you experienced a positive/negative impact through the speech therapy sessions, can you give an example of this? **How did you feel about your voice after starting speech therapy?** **How do you currently feel about your voice compared to when you started speech therapy?** **How would you like your voice to sound?** Additional questions: - Can you tell us something about the aspects that you would like to change? - You told me your voice sounds good, what does good mean to you? - What makes your voice sound good to you? - Which aspects are important in this? **What have you learned after the sessions?** **How did your environment react to the change in your voice?** Additional questions: - You mentioned that person X reacted in such a way, how did you experience this? - You mentioned that person X reacted in such a way, was his/her/their reaction important to you? - Do you experience a conflict between how your voice sounds and how others think your voice sounds?
**Speech therapy: effects and expectations**	**What expectations did you have about speech therapy?** Additional questions: - You said that the sessions did not meet your expectations, which aspects are important? - You said the sessions met your expectations, what does that mean to you? - You indicated that you still need sessions to adjust your voice, what would these entail? - What things did you miss in the sessions?
**Communicating with others, psychosocial impact**	**How do you experience communicating with others?** **How do you experience applying the learned techniques in daily life?** **Which techniques do you find the most difficult/easiest?** **Which techniques do you think are most important to sound feminine?** Additional questions: - You told me you feel (X), can you tell us more about this? - How do you experience communicating with people in your close environment? ⇒ How do you experience communicating at home /with your family? ⇒ How do you experience communicating with family? ⇒ How do you experience communicating with friends? - How do you experience communicating at work? - How do you experience communicating in public? - How do you experience communication when exercising hobbies? - You say that communication with X is difficult for you, can you tell us more about that? ⇒ Are there other people you experience this with? ⇒ How do you feel about this? - You say that you find it difficult to communicate in situation X, can you tell us more about that? ⇒ Are there any other situations where you experience this? ⇒ How do you feel about this? - You say that you experience certain communication difficulties, what impact do these communication difficulties have on your daily life?
**Misgendering**	**You mentioned that others address you as a man, how do you feel about this?** Additional questions: - You mentioned that you were addressed incorrectly on the phone, how did you feel about this? - What feelings do you experience when your communication partners do not perceive your voice as feminine? - How do you experience the way others address you? - Can you give an example? - You mentioned that in situation X you are addressed as a man, do you experience this in other situations as well? - Can you tell us more about this situation?
**Mental impact**	**What impact does your voice have on your mood?** **What impact did the sessions have on your self-confidence?** Additional question: - What feelings do you experience when you are confronted with X (describing the difficulties the person described)?
**Summary**	- We talked about X, X,.. and about X (summarizing the most important topics of conversation). X, X,… and X were important to you. Did I understand this correctly? Is it correct to conclude that you experienced an impact from X, X and X? - Would you like to add something to this? - Do you have any questions at this point?
**Closing and thanking**	

## Appendix D

**Table A3:** Table with Themes, Subthemes and Selected Quotes.

Themes	Subthemes	Selected Quotes
**Therapy experiences**	/	“***Especially** the part with the clear speech exercises, it was always 1 topic per session and then actually that last session was everything together, and that was all quite short together. A bit too difficult, I had also practiced the other (techniques) a bit, but only practicing one item at a time and then having to apply it again after 5 weeks and add the last one, was quite fast. Way too many (techniques). I thought the pitch part was a good tempo, I thought that was better*.” *(Participant 12)* “***I** sometimes had the feeling that it was going a bit fast at times. But it was good that most themes got their own session at the very least. Uh yes, it’s also the fact of, I have no experience with music or with voice in any other way, uhm, so for me that was all new information, uhm and because of that I sometimes had the feeling, yes, that it sometimes went quite fast or I found it difficult to hear correctly, how a certain technique affected the sound of my voice*.” *(Participant 3)*
	Voice changes	“***Pretty** low, because I know my voice is pretty low. Eh and I’ve always had problems with that so I kind of had the idea of speech therapy, I wanted to try it, but personally I had little expectations. That had nothing to do with whether it was speech therapy or not, rather because I know I have a low voice*.” *(Participant 11)* “***I** also don’t need to have the highest, not the most ‘fem’ voice that… Uh if I really wanted to I probably could, I do have the voice range for it, but it’s not really necessary for me. I already have something like, I’m where I want to be, and that was actually what I wanted, actually more than I wanted from the study*.” *(Participant 12)* “***So** at the end of the sessions I was really impressed by the big difference between before and after. I actually came in with pretty low expectations and I came out with very satisfied results*.” *(Participant 5)* “***Ehm**, yes, since I haven’t practiced much on myself yet, that hasn’t come up much yet, but that’s up to me. Not on the study, that’s just myself, who was under too much stress*.” *(Participant 1)*
	Difficulties in the training	“***And** if I notice that I forget to apply a technique in a conversation, I can also think about it for a while that I forgot to apply it. But then I just think, next time we’ll do it better.*” *(Participant 7)* “***Uhm**, well, to be honest I try to use it as a kind of mask for my low voice, so if I’m not hoarse then I actually have a very low voice, but if my voice is as hoarse as it is now then I use that in actually to cover up my low (voice), so yes, so for example now with a relative, in-laws, and I say there’s something wrong with my voice, and then she says yes that’s normal, but that’s actually to cover up that I have a low voice*.” *(Participant 11)*
	Relationship with the therapist	“***That**’s something where the therapist really helps, that you stay less focused on that bigger (goal), because that, that’s a bit like you want to run a marathon when you’re at a point where you can’t even run 5 km. There are still many intermediate steps to take and if you can’t see them it feels very intimidating and very unreachable.*” *(Participant 3)*
	Home exercises	“***Uh**, yeah, I just forget to do them that’s my problem. It’s just, I’ve already got so many other things to do that I’m just not going to get around to it I guess.*” *(Participant 1)* “***In** the beginning, because, yeah talking with lip spreading, yeah and with that forward tongue, that, I don’t know, that was exactly the same all the time in that bundle (of exercises), then I didn’t really think that…, that was so very repetitive and always the same and I wanted to bring a little more variation in that so I tried to practice that more by simply applying it more in daily life, for example by going for a walk with my brother and then trying to pay attention when I was talking*.” *(Participant 2)*
**Communication**	/	“***Yes**, I may feel happier because I don’t have to hold back. For example those phone calls, instead of an email that I can now really call directly and that I don’t feel bad about it. That is a bit of happiness <<laughs>>. So actually before the therapy I didn’t really have a fear of speaking in daily life, I wasn’t really afraid of it, but they had to see me. And now I dare to have more and more conversations without them seeing me. Like in the past when a friend who called me or something, I declined it and sent a message instead, that was so bad. And now I’m calling right away. That’s a difference*.” *(Participant 7)*
	Priority of the voice	“***It** is certainly not a frustration of mine, in the end I feel good, I have lived in hell and now I enjoy life. The voice is then, I’ll just say ‘surplus’. But it is certainly not a must.*” *(Participant 9)*
	Use of the new voice after therapy	“***And** now just the other day somebody said, “Say something in your old voice again, because I can’t remember.” And that didn’t work, I couldn’t sound the way I used to. It has really become such a habit, even to the kids and everyone who knows me, I just keep using a higher pitch*.” *(Participant 6)*
**Impact on mental health**	/	“***I** don’t actually know. I do not know. Because there are ten, twenty other things that also play a role that I really just don’t know anymore. My voice probably helped a lot with that, but (..) yes, I think it’s difficult to estimate how big the share was*.” *(Participant 1)* “***Um**, it has two parts. One, feeling good in what I am and I’ve always had that. I think my voice is more beautiful now than before, but I also thought my voice was beautiful then, it just didn’t quite match what I was and how I felt. Because I was different then than I am now. But on the other hand, voice is also something that is judged a lot, one of the first things people see/hear about you and one of the first things people give a first impression, and if you can make that first impression positive that is a boost for yourself as well. And do you feel better about that, especially if it also has a gender-affirming effect*.” *(Participant 12)* “***Uh**, I don’t know, sometimes that gives me a hopeless feeling that I have the impression that I am still very far away, if I can compare it with an analogy it is exactly that as a child you are playing with blocks and then that after a lot of training you can place three blocks on top of each other, but when you see that other people can immediately place ten blocks on each other, then you are not happy at all with the progress you have made of putting those three blocks together. Then you can only focus on ‘I’m really not quite sure what I would like to have’. And yes, that’s how it feels*.” *(Participant 2)* “***On** the one hand I may have the impression that I have become a bit more insecure because I don’t know what I sound like at all, but I think that’s because I used to know I sound like a man and then I don’t care anymore and I was also less involved with it so, because I’m more involved with it now, I’m going to worry about it a little more often. Then on the other hand, yes then I also feel better that people think they think I sound more feminine, or more in between, and um, yes I don’t know, in that respect I think I should actually feel more confident, but I don’t always feel that way*.” *(Participant 2)*
	Misgendering	“***Uh** (..) yes, so (...) it’s a bit of frustration, a bit of sadness, so what the feeling of, yes, falling short, yes, partly with that I’ve been working on it for a while, is it so a bit like, will I never get there, yes. Yeah, kind of like that kind of feeling*.” *(Participant 3)* “***Bad** feelings, I feel really really bad about that. Yes, it is, yes, how would I say, a knife you get in your own back, that hurts a lot. That hurts a lot*.” *(Participant 4)* “***I** think that’s also because people are also a bit more free in their gender expression these days, euh, which sometimes makes people think ah that’s just someone who is a very feminine man or something. And uh, which is also positive on one side. So I’m like, as long as it’s said kindly, and like, ‘oh sorry’, I don’t have a problem with that. It’s not something I think about afterwards or anything, it’s not something that invalidates me*.” *(Participant 12)*
**External factors associated with the outcome**	/	*N/A*
